# A miR-30 Guided Molecular Profiling of Canine Osteosarcoma and Extraskeletal Osteosarcoma Reveals Non-Seed Regulatory Divergence

**DOI:** 10.3390/cells14161279

**Published:** 2025-08-18

**Authors:** Gabriella Guelfi, Petronela Munteanu, Camilla Capaccia, Ilaria Porcellato, Elisabetta Manuali, Margherita Maranesi, Leonardo Leonardi

**Affiliations:** 1 Department of Veterinary Medicine, Università degli Studi di Perugia, 06126 Perugia, Italy; petronela_mun@yahoo.com (P.M.); camilla.capaccia@dottorandi.unipg.it (C.C.); ilariaporcellatodvm@gmail.com (I.P.); margherita.maranesi@unipg.it (M.M.); leonardo.leonardi@unipg.it (L.L.); 2 Laboratory of Comparative Veterinary Histopathology, Istituto Zooprofilattico dell’Umbria e delle Marche (IZSUM) Togo Rosati, 06126 Perugia, Italy; e.manuali@izsum.it

**Keywords:** canine osteosarcoma, extraskeletal osteosarcoma, miR-30 family, RUNX2, diagnostic biomarkers

## Abstract

Osteosarcoma (OS) and extraskeletal osteosarcoma (EOS) in dogs exhibit histological similarities but differ in anatomical origin, which poses a challenge to diagnostic accuracy. We adopted a marker-first strategy to enhance molecular classification by selecting RUNX2, KPNA2, and SATB2, three validated immunohistochemical (IHC) markers, as primary targets. Bioinformatic screening identified the miR-30 family as the only miRNA group predicted to coordinately regulate RUNX2, KPNA2, and SATB2, justifying its prioritization for expression analysis. RT-qPCR on FFPE tissues from 14 OS, 19 EOS, and 10 healthy controls revealed that miR-30a was significantly downregulated in OS and inversely correlated with RUNX2 nuclear expression, confirmed by IHC. MiR-30e also showed high diagnostic accuracy, while miR-30b and miR-30c distinguished EOS from OS. Non-seed interaction modeling (i.e., outside the canonical “seed” region, spanning nucleotides 2–8 of the miRNA) suggested divergent regulatory affinities within the PI3K/AKT/RUNX2 axis among miR-30 family members. MiR-30a and miR-30e exhibited the highest diagnostic power (LR^+^ 7.7 and 6.8, respectively), supporting their role as biomarkers. These results highlight a miR–30–centered regulatory axis with relevance for diagnosis and molecular stratification of canine osteogenic tumors.

## 1. Introduction

Skeletal osteosarcoma (OS) and extraskeletal osteosarcoma (EOS), though arising in different anatomical sites, often share remarkably similar pathological and molecular traits. These tumors exhibit rapid progression, frequent recurrence, highly metastatic potential, and resistance to conventional therapies [1,2]. Typically localized in the appendicular skeleton, OS affects large-breed dogs and young humans [3]. In contrast, EOS originates in soft tissues without skeletal attachment and, similarly to OS, can be characterized by the production of osteoid or immature bone [4]. Primary skeletal OS arises directly within bone tissue, differently from EOS, which originates in non-skeletal sites [5]. This distinction raises questions about the cellular origin of EOS and the molecular mechanisms that distinguish it from OS. Microscopically, OS and EOS are both composed of neoplastic osteoblasts with irregularly shaped nuclei, eosinophilic cytoplasm, and multinucleated osteoclast-like giant cells. The presence of osteoid is variable [6]. The histopathological overlap between OS and EOS complicates their distinction, requiring additional molecular and immunohistochemical markers for accurate classification [6,7,8].

On a molecular level, OS and EOS share deregulated pathways that affect osteoblastic differentiation, cell proliferation, and tumor invasion. These shared alterations have been identified, but the transcriptional, post-transcriptional, and epigenetic regulatory mechanisms driving them remain poorly understood [5,9,10]. Current immunohistochemical (IHC) and molecular analyses, including RUNX2 [5,11], KPNA2 [5,12], and SATB2 [13,14,15,16,17] as biomarkers, support the osteogenic origin of the tumor and assist in differentiating OS from morphologically similar neoplasms.

In humans and dogs, RUNX2 displays strong nuclear positivity in neoplastic cells, highlighting its central role in osteoblastic differentiation and its potential association with tumor aggressiveness [9,18]. This consistent expression supports the osteoblastic origin of the tumor, supporting the translational relevance of the canine model. Still, these approaches do not allow a definitive distinction between OS and EOS [5,9]. The lack of a clear molecular signature hampers the identification of reliable biomarkers.

In this context, microRNAs (miRNAs) have been identified as promising biomarkers because they play an important role in regulating gene expression in OS. Their potential to refine the molecular distinction between OS and EOS is particularly relevant in these tumors that share histological characteristics but differ in anatomical location and biological behavior [19].

Recent canine-specific studies have highlighted the relevance of miRNA expression profiles in osteosarcoma, demonstrating their association with key oncogenic pathways such as PI3K, mTOR, and cell cycle regulation [20], as well as their correlation with molecular subtype differentiation [21] and metastatic behavior [22]. These findings reinforce the value of miRNA-based strategies in the molecular classification and clinical management of canine OS. MiRNAs regulate tumor gene expression and microenvironmental interactions, acting locally and systemically via stable extracellular secretion [19,23,24,25,26]. Unlike mRNA, miRNAs exhibit higher stability and resistance to degradation through RNase, allowing them to persist longer in the extracellular environment. This property enhances their potential as diagnostic and prognostic biomarkers, as well as strengthening their role in intercellular signaling [27,28]. In addition, miRNAs actively modulate metastasis by regulating key processes such as cell migration, invasion, and colonization at distant sites [29]. Unlike mRNAs, miRNAs are ideal candidates for molecular profiling as they are not restricted to specific genes but target multiple genes of different signal networks.

In tumorigenesis, miRNAs function as oncogenes (oncomiRs) or tumor suppressors, depending on their targets and cellular context [30,31,32,33]. The miR-30 family, which includes miR-30a, miR-30b, miR-30c, miR-30d, and miR-30e, plays a key role in maintaining bone homeostasis by negatively regulating osteoblast differentiation. These miRNAs regulate osteoblast and osteoclast differentiation by targeting critical transcription factors such as RUNX2 [34] and SOX9 [35]. Additionally, miR-30 family target genes are mainly involved in key regulatory pathways, such as the p53 signaling pathway, which governs cell cycle control and apoptosis [36]; the Wnt signaling pathway, which influences cell growth and differentiation [37]; and the mTOR and PI3K/AKT pathways, which are crucial signaling pathways contributing to tumor growth, survival, and metastasis [38].

This study aims to elucidate the molecular classification of canine osteogenic tumors by clarifying the post-transcriptional regulatory mechanisms underlying OS and EOS. In this research, instead of performing miRNome-wide screening, we adopted a marker-guided strategy that leverages established phenotypical and immunohistochemical (IHC) markers RUNX2, KPNA2, and SATB2, which are commonly used in OS diagnosis, to identify microRNAs that may potentially regulate these targets. The miR-30 family was the only family predicted to bind all three markers, thus representing a candidate regulatory axis underlying the osteogenic molecular identity of the tumor. QPCR-based expression analysis of miR-30 family members was performed on FFPE tissue samples from canine OS, EOS, and healthy controls. Following preliminary histological examination and RUNX2 IHC assessment, we evaluated a diagnostic cutoff threshold for miR-30 family members to refine molecular classification, aiming to improve diagnostic specificity and support clinical management, including potential early detection via biopsy or circulating biomarkers.

## 2. Materials and Methods

### 2.1. Experimental Workflow

**Figure 1:**
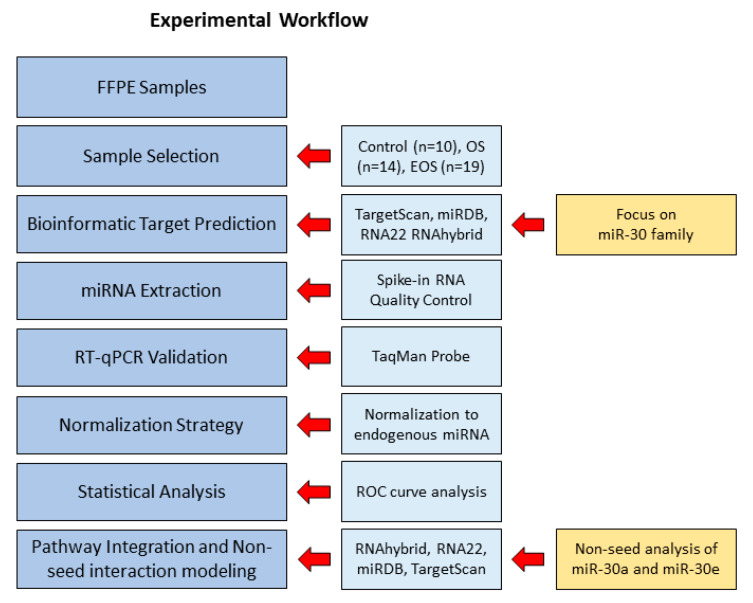
FFPE samples from canine OS, EOS, and CTRL were selected and processed for miRNA profiling. Bioinformatic prediction of miR-30 family targets (with a specific focus on miR-30 members) was followed by RNA extraction with spike-in quality control, reverse transcription, and quantitative PCR (qPCR). Normalization was based on the most stable endogenous miRNA. Statistical analysis included ROC curve evaluation for diagnostic performance. Pathway integration and non-seed interaction modeling included targeted analysis of miR-30a and miR-30e, and these analyses were performed using RNAhybrid, RNA22, miRDB, and TargetScan.

To investigate the post-transcriptional regulation of key diagnostic markers in canine osteogenic tumors, we first selected RUNX2, KPNA2, and SATB2 based on their validated use in IHC diagnosis of OS and EOS. A bioinformatic screening was then performed to identify miRNAs capable of coordinately targeting these genes. Among all screened families, only the miR-30 family was predicted to regulate RUNX2, KPNA2, and SATB2, supporting its selection for further expression analysis. FFPE tissue samples from OS, EOS, and control (CTRL) dogs were processed for RNA extraction and quality assessment. MiRNA expression was analyzed by RT-qPCR and normalized using the most stable endogenous control identified via RefFinder. Statistical analysis and ROC curve-based cutoff determination were performed to evaluate the diagnostic potential of miR-30 family members. Pathway mapping and duplex simulations were conducted to explore seed and non-seed regulatory mechanisms (Figure 1).

### 2.2. Case Selection and Inclusion Criteria

The present study was conducted on a total of 43 formalin-fixed, paraffin-embedded (FFPE) canine tissue samples, retrieved from the archives of the Veterinary Pathology Section of the Department of Veterinary Medicine (University of Perugia) and the Istituto Zooprofilattico Sperimentale dell’Umbria e delle Marche (IZSUM, Togo Rosati, Perugia). The samples were obtained as surgical biopsies submitted for diagnostic histopathology between 2010 and 2025. A preliminary power analysis, based on the observed differences among groups, indicated that a minimum of 12 subjects per group would provide ≥90% statistical power to detect significant differences. The study included 10 CTRL samples, 14 primary skeletal osteosarcoma (OS) cases, and 19 extraskeletal osteosarcoma (EOS) cases. Study inclusion was based on the following criteria: (i) histological diagnosis of OS or EOS, with immunohistochemical confirmation in uncertain cases; (ii) availability of neoplastic tissue with a surface area > 0.5 cm^2^ on the section; and (iii) clinical evidence of primary tumor origin. For EOS cases, the absence of skeletal involvement was confirmed by radiographic or computed tomography (CT) imaging to ensure an extraskeletal origin. Cases showing fixation artifacts upon histological examination were excluded. Clinical data, including breed, age, sex, tumor localization, and histological diagnosis, are summarized in Table 1.

### 2.3. Bioinformatics-Based Selection of RUNX2, KPNA2, and SATB2 miRNA-Target Interactions

Based on the diagnostic relevance of RUNX2, KPNA2, and SATB2 in canine OS and EOS, these genes were selected as primary targets. These genes have previously been identified as diagnostic markers for canine OS [5,13,14,15,16]. A bioinformatic screening was conducted to identify miRNAs potentially regulating them. Among all families analyzed, only the miR-30 family was consistently predicted to bind all three genes, supporting its selection for downstream expression analysis. Target prediction was performed using TargetScan [39], which identifies conserved binding sites within the 3’UTR regions, miRDB [40], which employs the MirTarget model, RNA22 [41], and RNAhybrid [42]. Gene annotations were validated through the HUGO Gene Nomenclature Committee (HGNC) [43], and tissue-specific expression patterns were retrieved from the Human Protein Atlas [44]. Functional enrichment analysis was conducted using miRPath v3.0 [45]. RUNX2, KPNA2, and SATB2 emerged as shared targets of all five miR-30 members (miR-30a to -30e) across at least three databases, with conserved binding sites located in their 3′UTRs (Table S1).

### 2.4. Seed Sequences of miR-30 Family

All members of the miR-30 family share the highly conserved seed sequence GUAAACA (nt 2–8), suggesting functional similarities and the potential to regulate overlapping gene targets. In contrast, non-seed regions (nt 9–22), primarily located in the 3′ portion of the mature sequences, may modulate target specificity and influence the regulation of distinct biological pathways (Table 2).

### 2.5. RNA Spike-In Quality Assessment

To evaluate miRNA extraction, cDNA synthesis, and qPCR performance from FFPE samples, spike-in controls ensured technical reproducibility and linearity. A 1 µL mix of UniSp2 and UniSp4 (QIAGEN CLC bio, Aarhus, Denmark) was added to the lysis buffer before RNA isolation to assess extraction efficiency and yield, following QIAGEN standard protocol. UniSp6 was incorporated during cDNA synthesis to evaluate synthesis efficiency and detect potential PCR inhibitors. In the qPCR phase, UniSp2, UniSp4, and UniSp6 were amplified and quantified using specific primer pairs as recommended by the manufacturer. This quality control approach allowed for comprehensive workflow monitoring, ensuring reliable gene expression analysis. The UniSp2 and UniSp4 RNA spike-in mix was designed so that UniSp2 is present at a concentration 100-fold higher than UniSp4.

### 2.6. MiRNA Isolation and Purification

Total RNA, including miRNAs, was extracted from FFPE tissue samples using the miRNeasy FFPE Kit (QIAGEN, Hilden, Germany) according to the manufacturer’s protocol. Briefly, FFPE sections were deparaffinized with xylene, followed by ethanol washes. The samples were then lysed in a proteinase K-containing buffer at 56 °C to digest proteins and release nucleic acids. To optimize RNA recovery and remove formalin-induced crosslinks, a DNase treatment step was performed. The RNA was then purified using silica membrane-based spin columns. For quality control, 1 µL of spike-in mix UniSp2 and UniSp4 (QIAGEN CLC bio, Aarhus, Denmark) was added. RNA quality and quantity were assessed using the Qubit Fluorometer 4 and the Qubit microRNA Assay Kit (Thermofisher Scientific, Kandel, Germany). The integrity and quality of RNA (RNA IQ) extracted from FFPE samples were assessed using the Qubit™ RNA IQ Assay Kit (Thermo Scientific, Waltham, MA, USA). The assay provides a qualitative estimation based on the relative proportion of intact double-stranded RNA to degraded single-stranded RNA. RNA samples were stored at −80 °C until further analysis.

### 2.7. RT-qPCR Analysis of miRNA Expression

cDNA was synthesized using the miRCURY LNA RT Kit (QIAGEN, Hilden, Germany). The reaction mixture included 5× miRCURY RT reaction buffer (2 µL), 10× miRCURY RT enzyme mix (1 µL), synthetic UniSp6 spike-in RNA (0.5 µL), 10 ng of total RNA, and RNase-free water to a final volume of 10 µL. The reaction was incubated at 42 °C for 60 min, followed by enzyme inactivation at 95 °C for 5 min. The synthesized cDNA was then stored at −20 °C until use. qPCR reactions were performed in a final volume of 10 µL using the miRCURY LNA SYBR Green PCR Kit (QIAGEN, Hilden, Germany), containing 5 µL of SYBR Green master mix, 3 µL of cDNA template (diluted 1:60), 1 µL of miRNA-specific LNA primer mix (Table 3), and RNase-free water to reach the final volume.

To assess RNA extraction efficiency and qPCR performance, synthetic spike-in control primers UniSp2, UniSp4, and UniSp6 were included in the qPCR mix. Amplification was carried out with an initial enzyme activation at 95 °C for 2 min, followed by 40 cycles of denaturation at 95 °C for 10 s and annealing/extension at 56 °C for 60 s, during which fluorescence acquisition was performed. Melting curve analysis (60–95 °C) was performed to assess amplification specificity. QPCR amplification was performed using a 96-well optical plate on a StepOnePlus™ Real-Time PCR System (Applied Biosystems, v2.3, Carlsbad, CA, USA). Each biological sample was analyzed in triplicate, and the mean quantification cycle (Cq) value was determined using StepOne Software (v2.3, Applied Biosystems). No-template controls (NTCs) were included in each run to confirm the absence of genomic DNA contamination. Amplification efficiency was assessed by calculating the slope of the standard curve using the formula Efficiency = 10^(–1/slope)^. PCR conditions were optimized to achieve an efficiency greater than 95%, and only reactions with values between 95% and 100% were considered for further analysis. All qPCR assays complied with the Minimum Information for Publication of Quantitative Real-Time PCR Experiments (MIQE) guidelines [48]. Normalized target miRNA expression levels were calculated using the Livak 2^−ΔCq^ method [49], with normalization against the most stable endogenous control (EC) miRNA (ΔCq = Cq_target − Cq_best EC miRNA). Relative miRNA expression was then determined using the 2^−ΔΔCq^ method, where the second Δ value represents the difference between the normalized expression of the sample and that of the experimental control [50]. The amplification and the results of interpreting spike-ins were performed according to Guelfi et al. [51].

### 2.8. RUNX2 Immunohistochemistry in OS, EOS, and CTRL Tissue

Section 5 μm thick were obtained from FFPE tissue blocks and mounted on poly-L-lysine-coated microscope slides. The slides were dewaxed, rehydrated, and subjected to heat-induced epitope unmasking in Tris-EDTA buffer (pH 9.0) in a microwave for 20 min. Immunohistochemical staining was performed using an anti-RUNX2 monoclonal antibody (Thermo Scientific, Santa Cruz, CA, USA, clone F-2; dilution 1:200), as previously described [9]. After a 2 h incubation at RT with the primary antibody, the slides were treated with an ABC detection ready-to-use kit (Abcam, Cambridge, UK) according to the manufacturer’s instructions. 3-amino-9-ethylcarbazole (AEC) was used as chromogen to reveal immunolabeling, and Carazzi’s hematoxylin was applied as a counterstain. Negative control sections were incubated with PBS instead of the primary antibody to confirm specificity; healthy tissues included in the study were used as additional negative controls. An immunohistochemical evaluation was performed by estimating the proportion of RUNX2-positive nuclei within the neoplastic population and assigning a percentage value (0–100%). Three high-power fields (400× magnification) were selected from the most representative tumor areas, and at least 500 tumor nuclei per case were counted. The percentage of RUNX2-positive nuclei was calculated as the ratio of immunolabeled nuclei to the total number of neoplastic nuclei examined. Immunoreactivity of nuclear staining was determined semi-quantitatively by evaluating the extent of staining (percentage of positive cells) and labeling intensity [9]. Nuclear labeling was scored on a 0 to 4 scale evaluating the percentage of positive nuclei as follows: score 0 indicated no nuclear labeling, score 1 indicated minimal labeling 1–15% of nuclei, score 2 indicated 16% to 40% positive nuclei, score 3 indicated 41–75% nuclei, and score 4 indicated a strong positive labeling of 76% to 100% of nuclei. Staining intensity was attributed as follows: 0—no staining, 1—weak intensity, 2—mild intensity, and 3—strong intensity.

### 2.9. Statistical Analysis

Receiver operating characteristic (ROC) curves, area under the curve (AUC), sensitivity, and specificity were used to assess the diagnostic performance of each miRNA. Differences in 2^−ΔCq^ normalized miRNA expression and in 2^−ΔΔCq^ relative miRNA expression between groups were evaluated using an unpaired Student’s *t*-test. A *p*-value < 0.05 was considered statistically significant. All statistical analyses and graphical outputs were generated using GraphPad Prism 8 (GraphPad Software, Inc., San Diego, CA, USA). In this last method, the calibrator group (CTRL) is mathematically assigned a relative expression value of 1 (since 2^0^ = 1), and all other samples are expressed in relation to this baseline.

## 3. Results

### 3.1. Histological Diagnosis and IHC Evaluation of RUNX2

**Figure 2:**
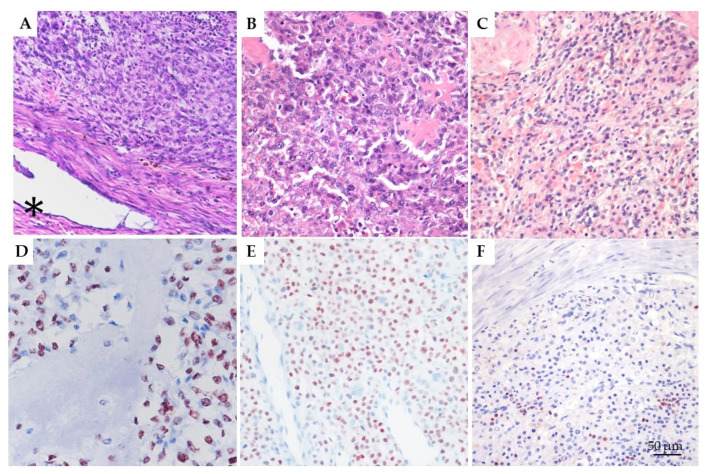
IHC evaluation of RUNX2: (**A**) Extraskeletal osteoblastic osteosarcoma (mammary gland) with minimal osteoid production. Residual ducts of the mammary gland can be seen around the tumor (asterisk; 400×, HE). (**B**) Skeletal osteoblastic osteosarcoma (humerus) with moderate osteoid production (400×, HE). (**C**) Healthy spleen, one of the negative controls selected for the study (400×, HE). (**D**) Extraskeletal osteoblastic osteosarcoma (mammary gland) with abundant osteoid production. RUNX2 shows stronger immunolabeling near areas of osteoid deposition (400×, AEC and hematoxylin). (**E**) Skeletal osteoblastic osteosarcoma (humerus) with moderate osteoid production, displaying more than 60% RUNX2 positivity (400×, AEC and hematoxylin). (**F**) Healthy spleen, one of the negative controls selected for the study. RUNX2 expression is also observed in occasional lymphocytes, likely T cells, as reported in previous studies [52] (400×, AEC and hematoxylin). Scale bars: 50 µm (**A**–**F**). Residual ducts of the mammary gland, indicated by *, can be seen around the tumor (400×, HE).

Histological re-evaluation was performed separately by three pathologists (LL, IP, and EM). RUNX2 was observed in all the tested tumors, with a variable percentage of positive nuclei. Immunolabeling was strong. particularly in the neoplastic cells surrounding osteoid matrix (Figure 2).

Immunohistochemical data (% of RUNX2-positive nuclei, semi-quantitative score, and staining intensity) were analyzed across the three groups, OS, EOS, and CTRL (Table 4).

Due to non-normal distribution of most variables (Shapiro–Wilk test, *p* < 0.05), non-parametric tests were employed. The Kruskal–Wallis test revealed significant differences among groups for all parameters: % Labeled Cells (H = 19.50, *p* < 0.0001), Score (H = 21.51, *p* < 0.0001), and Intensity (H = 22.85, *p* < 0.00001). Post hoc pairwise comparisons using the Mann–Whitney U test showed no significant differences between OS and EOS (*p* > 0.05), while both tumor groups differed significantly from CTRL (*p* < 0.0001 for all variables). Accordingly, superscript letters were applied to individual values in the table to denote statistical groupings: a for CTRL, b for both OS and EOS. Although the three parameters are biologically related, they were analyzed independently, following a standard approach in immunohistochemical studies, as also applied in comparable veterinary pathology literature [9].

### 3.2. RNA Evaluation and Reference Gene Selection

The RNA 260/280 ratio, ranging from 1.8 to 2.0, confirmed high RNA purity, while total RNA yields were consistent across samples, with no significant variations observed. RNA samples showed good integrity, as confirmed by Qubit™ RNA IQ assay, with RNA IQ values ranging between 7 and 10, indicating suitability for downstream analyses. The Cq difference of approximately 7 cycles between UniSp2 and UniSp4 (21 and 28 cycles, respectively) confirmed both RNA integrity and efficient extraction. Additionally, UniSp6 amplification at approximately 19 cycles validated cDNA synthesis and qPCR performance, ensuring the reliability of gene expression analysis. To minimize experimental variability due to differences in RNA input, qPCR data were normalized using the most stable EC miRNA. The optimal EC miRNA was identified through four mathematical approaches: Delta Ct [53], BestKeeper [54], NormFinder [55], and GeNorm [56]. The RefFinder integration tool was then employed to compare and rank the results from these algorithms [57], identifying miR-103a-3p as the most suitable EC miRNA for qPCR normalization (Table 5).

### 3.3. Expression Profiling of miR-30 Family Members in CTRL, OS, and EOS Samples

**Figure 3:**
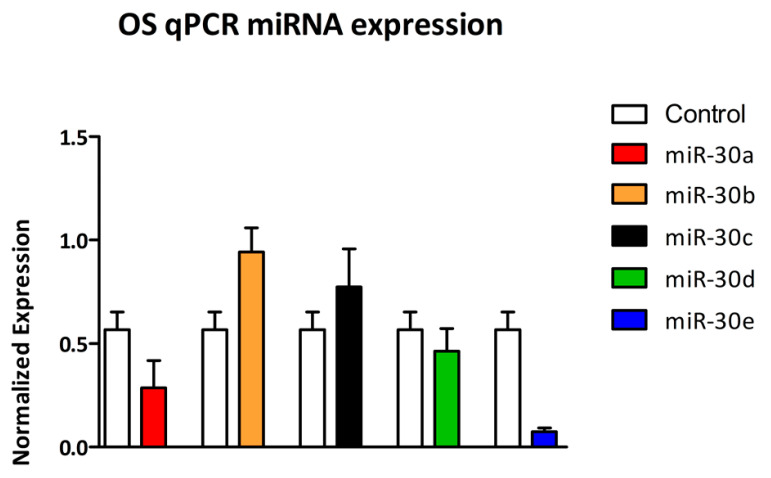
Normalized expression levels (2^−ΔCq^) of miR-30a, miR-30b, miR-30c, miR-30d, and miR-30e in CTRL versus OS groups. Expression is measured using the 2^−ΔCq^ method. Bars represent mean ± SEM. The expression levels of miR-30a, miR-30b, and miR-30e exhibit statistically significant differences. Conversely, miR-30c and miR-30d display no statistically significant difference between the two groups (*p* > 0.05).

Among the five members of the miR-30 family, distinct expression patterns were observed across the three experimental groups. Compared to CTRL, miR-30a, miR-30b, and miR-30e levels were significantly reduced in OS samples (*p* < 0.05, *p* < 0.05, and *p* < 0.01, respectively). In contrast, miR-30c and miR-30d showed no statistically significant differences between groups (*p* > 0.05) (Figure 3).

**Figure 4:**
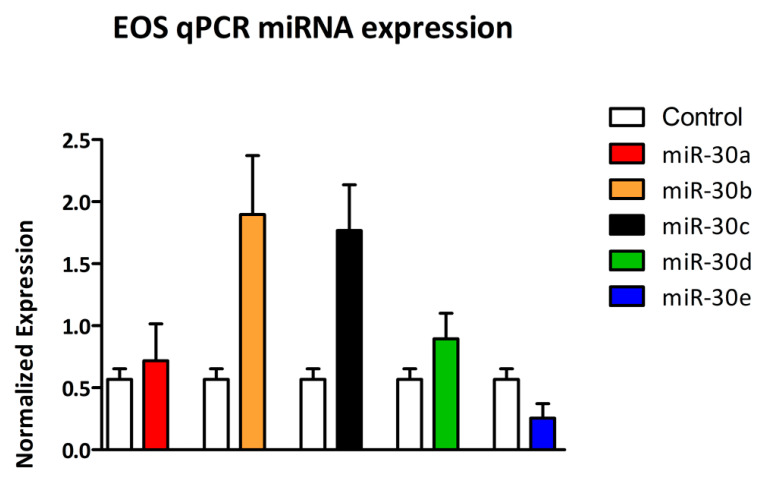
Normalized expression levels (2^−ΔCq^) of miR-30 family members in CTRL versus EOS groups. MiRNA expression is measured using the 2^−ΔCq^ method. Bars represent mean ± SEM. The expression levels of miR-30c and miR-30e exhibit statistically significant differences. In contrast, statistically significant differences are not observed for miR-30a, miR-30b, and miR-30d between the two groups (*p* > 0.05).

Expression analysis of the miR-30 family between CTRL and EOS groups revealed a significant upregulation of miR-30c in EOS samples (*p* < 0.05). In contrast, miR-30e showed the opposite trend: its expression was significantly downregulated in the EOS group (*p* < 0.05). No statistically significant differences were observed for miR-30a, miR-30b, and miR-30d between the two groups (*p* > 0.05) (Figure 4).

**Figure 5:**
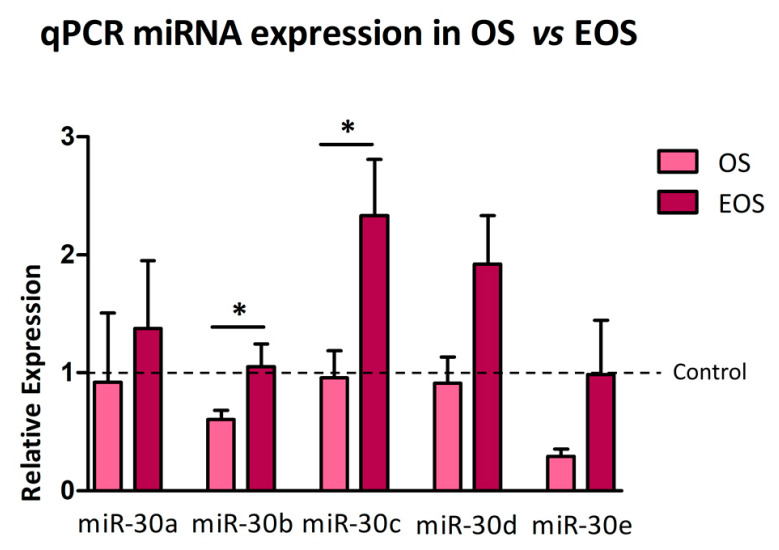
Fold changes (2^−ΔΔCq^) of miR-30a, miR-30b, miR-30c, miR-30d, and miR-30e in OS versus EOS samples. Expression values are shown relative to the CTRL group, set to 1 (dashed line). Values above or below 1 indicate upregulation or downregulation, respectively, compared to CTRL. Only miR-30b and miR-30c exhibit statistically significant differential expression between OS (light red) and EOS (dark red), with *p* < 0.05 (*), whereas the other miR-30 members show no significant differences (*p* > 0.05). Data are presented as mean ± SEM.

Quantitative comparison of miRNA expression between OS and EOS groups was performed using the 2^−ΔΔCq^ method, which calculates relative expression levels normalized to CTRL (set as fold change = 1). miR-30b and miR-30c exhibited significantly higher expression in OS compared to EOS (*p* < 0.05) when comparing fold change values. In contrast, no significant differences were found for miR-30a, miR-30d, or miR-30e between the two tumor types (*p* > 0.05) (Figure 5 and Table S2).

### 3.4. Cutoff Selection for Diagnostic Specificity

To optimize the diagnostic performance of the miR-30 family, receiver operating characteristic (ROC) curve analysis was conducted to identify the most informative cutoff values. The optimal threshold for each miRNA was selected based on the highest positive likelihood ratio (LR^+^), which reflects the test’s ability to correctly identify affected cases while minimizing false positives. The choice of using LR^+^ as a selection criterion was guided by its recognized clinical relevance in diagnostic accuracy studies, and the interpretive thresholds applied in this study align with widely accepted standards in evidence-based test evaluation [58,59]. ROC curve analysis was applied to miR-30a, miR-30b, miR-30c, miR-30d, and miR-30e to evaluate diagnostic performance in distinguishing CTRL subjects from OS and EOS cases. The values of the area under the curve (AUC), optimal cutoff, sensitivity (%), specificity (%), and LR^+^ are summarized in Table 6.

**Figure 6:**
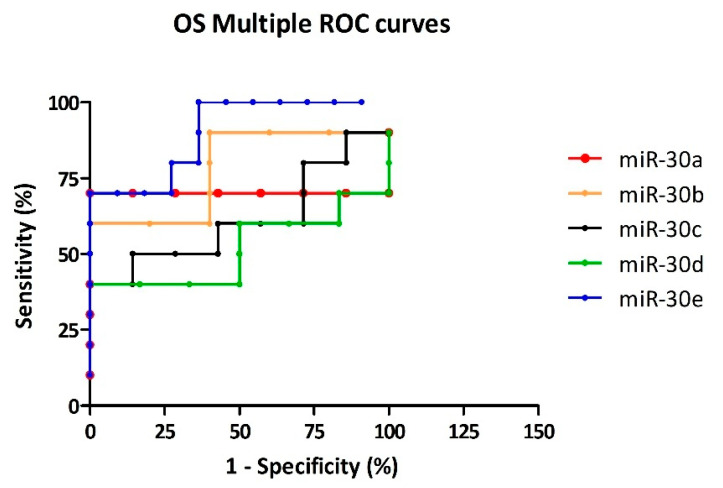
Multiple ROC curves comparing the diagnostic performance of miR-30 family members in distinguishing OS patients from CTRL. Curves represent the trade-off between sensitivity and specificity at optimal cutoff values, selected based on the highest LR^+^. The colored lines represent the ROC curves of the individual markers: miR-30a (red), miR-30b (orange), miR-30c (black), miR-30d (green), and miR-30e (blue). MiR-30e exhibits the highest diagnostic accuracy among the tested markers.

ROC analysis of OS revealed that miR-30e achieved the best diagnostic performance (AUC 0.900; sensitivity 70%; specificity 90.91%; LR^+^ 7.7). This was followed by miR-30a (AUC 0.714; sensitivity 71.43%; specificity 85.71%; LR^+^ 5), miR-30b (AUC 0.780; sensitivity 60%; specificity 80%; LR^+^ 3), miR-30c (AUC 0.614; sensitivity 50%; specificity 85.71%; LR^+^ 3.5), and miR-30d (AUC 0.518; sensitivity 40%; specificity 83.33%; LR^+^ 2.4), all showing acceptable performance with moderate LR^+^ values (Figure 6).

**Figure 7:**
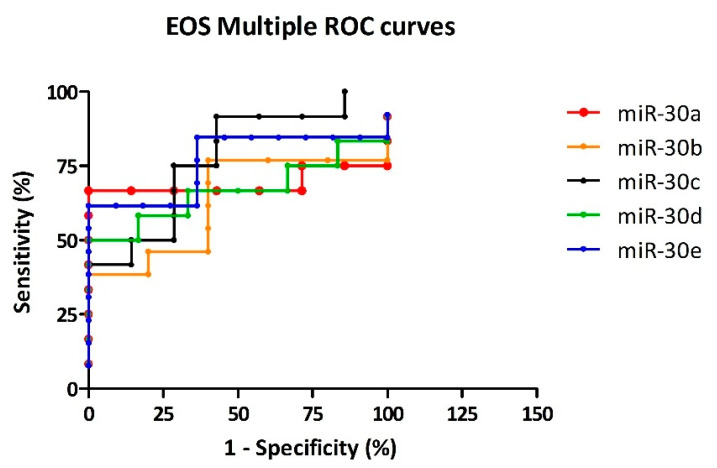
Multiple ROC curves illustrating the diagnostic ability of miR-30 family members to distinguish EOS cases from CTRL. Each curve reflects the sensitivity–specificity relationship at the optimal threshold, defined by the highest LR^+^. The five miRNAs are color-coded: miR-30a (red), miR-30b (orange), miR-30c (black), miR-30d (green), and miR-30e (blue). Among them, miR-30e exhibits the highest overall sensitivity and specificity, indicating superior diagnostic performance in EOS.

ROC analysis of EOS revealed that miR-30e achieved the best diagnostic performance (AUC 0.762; sensitivity 61.54%; specificity 90.91%; LR^+^ 6.8). This was followed by miR-30a (AUC 0.691; sensitivity 66.67%; specificity 85.71%; LR^+^ 4.7), miR-30b (AUC 0.631; sensitivity 46.15%; specificity 80%; LR^+^ 2.3), miR-30c (AUC 0.774; sensitivity 50%; specificity 85.71%; LR^+^ 3.5), and miR-30d (AUC 0.667; sensitivity 58.33%; specificity 83.33%; LR^+^ 3.5), all showing acceptable diagnostic utility (Figure 7).

### 3.5. Non-Seed Bioinformatic Mapping of miR-30a and 30e in Canine OS and EOS Signaling Pathways

To investigate the molecular mechanisms underlying the diagnostic specificity of miR-30a and miR-30e in distinguishing OS and EOS, we conducted a targeted bioinformatic analysis of miRNA–mRNA interactions, with particular attention to contributions beyond the seed region. Although the shared seed sequence of the miR-30 family enables recognition of common targets such as RUNX2 (see also “Bioinformatics-Based Selection of miRNA–Target Interactions”), the analysis revealed that distinct non-seed profiles of miR-30a and miR-30e confer differential binding affinities to RUNX2 and to additional mRNA targets. Based on their “Good” diagnostic performance (LR^+^ ≥ 5) for OS (miR-30a and miR-30e) and EOS (miR-30e only), these two miRNAs were selected for in-depth analysis using RNAhybrid [42], RNA22 [41], TargetScan [36], and miRDB [37]. These tools enabled the prediction of candidate target genes and their associated pathways, simulation of duplex formation, and assessment of the thermodynamic feasibility of non-canonical interactions [61]. RNAhybrid predicted a relatively more stable interaction between miR-30a and the RUNX2 3′UTR compared to miR-30e (ΔG = –26.0 kcal/mol vs. −25.6 kcal/mol), with base-pairing differences confined to the non-seed region. This finding may contribute to a molecular rationale for the selective downregulation of miR-30a observed in OS and its potential role in the post-transcriptional regulation of RUNX2 in osteosarcoma pathogenesis.

**Figure 8:**
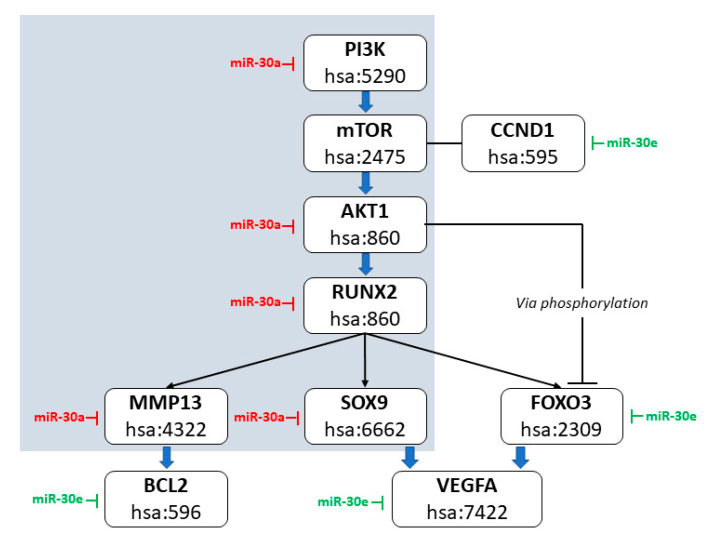
Schematic representation of the PI3K/AKT/mTOR–RUNX2 signaling axis and its transcriptional downstream targets, highlighting predicted miRNA-mediated regulatory interactions. The gray-shaded area delimits the predicted miR-30a–regulated axis, comprising key signaling components: PI3K (hsa:5290), mTOR (hsa:2475), AKT1 (hsa:860), and the transcription factor RUNX2 (hsa:860). Each box shows the gene name and the corresponding KEGG human gene identifier. Red lines represent predicted miR-30a inhibitory interactions, while green lines indicate miR-30e-mediated repression. MiR-30a is predicted to directly target PI3K, mTOR, AKT1, RUNX2, as well as the downstream effectors MMP13 (hsa:4322) and SOX9 (hsa:6662). MiR-30e is predicted to repress FOXO3 (hsa:2309), VEGFA (hsa:7422), and BCL2 (hsa:596). Arrow from mTOR to CCND1 (hsa:595) indicates the known role of mTORC1 in promoting cyclin D1 expression, thus contributing to cell cycle progression. FOXO3 is modulated transcriptionally via RUNX2 and post-translationally via AKT1-mediated phosphorylation, which leads to its cytoplasmic sequestration and functional inhibition. All interactions are based on in silico predictions from RNAhybrid, RNA22, TargetScan, and miRDB, and align with known molecular interactions within the KEGG PI3K-Akt pathway (hsa04151). Red labels indicate direct interactions of the miR-30 family within the core pathway (grey area), while green labels indicate additional predicted regulatory interactions.

To further investigate the biological implications of these findings, we mapped the predicted targets of miR-30a and miR-30e along the PI3K–AKT–RUNX2 signaling axis, which is functionally relevant in osteogenic differentiation, proliferation, and survival according to the Kyoto Encyclopedia of Genes and Genomes (KEGG) database (hsa04151). While RUNX2 is not explicitly included in this canonical pathway, previous studies support its transcriptional activation downstream of AKT1 phosphorylation, particularly in osteogenic and oncogenic contexts [62,63,64]. Among the AKT isoforms, AKT1 is recognized as functionally dominant in osteoblast-like cells and osteosarcoma. Bioinformatic predictions (TargetScan, miRDB, RNA22) indicate that miR-30a represses both AKT1 and RUNX2, supporting a dual-layered regulatory mechanism. miR-30a may thus inhibit RUNX2 both directly and indirectly via AKT1 downregulation, with all predicted interaction sites residing in the non-seed region. Additional predicted targets of miR-30a and miR-30e within this pathway include PIK3CA, mTOR, FOXO3, MMP13, SOX9, BCL2, VEGFA, and CCND1, all of which have been previously associated with osteosarcoma pathogenesis. These genes contribute to processes such as stemness maintenance, matrix remodeling, and resistance to apoptosis. A KEGG-adapted diagram summarizes the RUNX2 regulatory signal (Figure 8).

## 4. Discussion

The persistent lack of reliable biomarkers for canine OS and EOS prompted a biologically guided prioritization strategy centered on transcriptional markers and post-transcriptional miRNA regulation.

MiR-30a has been validated in human OS for its diagnostic and prognostic relevance in both bone tissue and plasma. Its expression inversely correlates with metastasis and survival, reinforcing its tumor-suppressive role and suggesting potential translational significance in canine OS [65,66,67]. This study adopted a marker-first strategy by selecting RUNX2, KPNA2, and SATB2 as diagnostic anchors based on their validated IHC relevance in OS. Rather than mapping downstream pathways—where no shared regulatory node emerges—we prioritized the identification of miRNAs predicted to coordinately regulate all three markers. This approach led to the miR-30 family, representing a convergent post-transcriptional axis that would have been overlooked by conventional pathway-based selection. Through this framework, the miR-30 family emerged as the sole candidate predicted to coordinately regulate three of the four markers (RUNX2, KPNA2, and SATB2), supporting its selection as a biologically driven diagnostic axis. Within this regulatory family, individual members displayed distinct diagnostic patterns. MiR-30e and miR-30a demonstrated the highest diagnostic performance (LR^+^ 7.7 and 5.0, respectively), qualifying as “good” markers for OS detection. However, their expression levels did not significantly differ between OS and EOS, indicating utility in disease identification rather than subtype discrimination. Consistent with this mechanism, miR-30a was significantly downregulated in OS compared to healthy CTRL, potentially enabling derepression of RUNX2 and promoting cancer stem cell maintenance [8]. Importantly, miR-30a downregulation was not observed in EOS, suggesting that the miR-30a–RUNX2 axis may be selectively engaged in OS. IHC analysis demonstrated nuclear RUNX2 expression in both OS and EOS, observed in central tumor cores and invasive tumor areas, supporting its role in transcriptionally active tumor cells [9]. These protein-level findings align with miR-30a downregulation and reinforce the proposed miRNA–RUNX2 regulatory axis. This differential behavior might reflect a distinct transcriptional landscape or the absence of specific damage signals required to activate this axis in EOS. Despite its mechanistic relevance, miR-30a did not discriminate between tumor subtypes, reinforcing the idea that its role pertains more to tumor biology than classification. Conversely, miR-30b and miR-30c, though exhibiting lower diagnostic power (LR^+^ 3.0 and 3.5), were the only members showing statistically significant differential expression between OS and EOS (*p* < 0.05), suggesting a potential role in subtype stratification. These findings highlight how miRNAs with modest classification accuracy may still capture biologically meaningful distinctions, warranting their consideration for molecular taxonomy or therapeutic targeting.

All miR-30 members share a conserved seed region (nt 2–8); however, the divergence in the non-seed region (nt 9–22) confers target specificity [68]. Notably, a single nucleotide difference at position 13 distinguishes miR-30a (G, guanine) from miR-30e (U, uracil). Despite being subtle, this variation significantly affects the stability and specificity of mRNA binding [69]. RNAhybrid predictions indicated that miR-30a forms a more thermodynamically stable duplex with RUNX2 (ΔG = −22.1 kcal/mol) than miR-30e (ΔG = −18.8 kcal/mol), with enhanced binding driven by non-seed complementarity. These differences were reflected in sequences such as AUCCUCGACUGGAAG (miR-30a) vs. AUCCUUGACUGGAAG (miR-30e) [69]. This specificity was also observed with osteogenic targets such as SOX9 and CD44, suggesting a broader regulatory role in stemness and differentiation [30,35].

While all three markers were initially prioritized for their diagnostic relevance, RUNX2 progressively gained prominence due to its consistent overexpression and strong inverse correlation with miR-30a levels. This observation, supported by pathway predictions and existing literature, led us to investigate the PI3K/AKT cascade as a mechanistic framework underpinning RUNX2-mediated oncogenic signaling. The PI3K/AKT signaling cascade is critically involved in modulating transcriptional networks that support oncogenesis in OS [70]. Within this pathway, AKT1 operates as a pivotal upstream regulator of the transcription factor RUNX2, a key effector implicated in tumor progression and species, highlighting its potential as a translational target. Post-transcriptional regulation of AKT1 by miR-30a has been shown to significantly reduce its expression, resulting in downmodulation of RUNX2 activity and suppression metastatic dissemination [63]. The integrity of this regulatory axis is maintained across of tumor growth [71,72,73,74,75]. This interaction illustrates a functional axis where miR-30a exerts tumor-suppressive effects by directly targeting AKT1, influencing the downstream transcriptional landscape. AKT1 has also been shown to be essential for RUNX2 activation and nuclear localization, thereby sustaining oncogenic transcriptional activity in OS cells [63]. Pharmacological inhibition of AKT1 not only diminishes RUNX2 expression but also impairs the maintenance of cancer stem-like cell populations, indicating therapeutic sensitivity of this regulatory mechanism [63,76]. This pathway’s relevance has been corroborated in canine OS models, where the conservation of PI3K/AKT–RUNX2 signaling supports its use in translational and preclinical research [76,77]. In particular, recent canine-specific transcriptomic studies have confirmed activation of PI3K and mTOR pathways in osteosarcoma, reinforcing the biological relevance of this regulatory axis in the canine setting [20,21]. These insights strengthen the hypothesis that the miR-30–AKT1–RUNX2 axis operates within conserved oncogenic networks in the dog and support its investigation as both a diagnostic marker and a potential therapeutic target. Overall, this positions the AKT1–RUNX2 signaling linkage as a conserved and actionable target in OS biology. The modulatory role of miR-30a on this axis not only offers insight into tumor suppression mechanisms but also opens new perspectives for the development of precision-based therapeutic interventions.

Although SATB2 and KPNA2 were included among the predicted targets of the miR-30 family, their expression was not experimentally assessed in this study. These targets were selected based on their diagnostic relevance and bioinformatic consistency, and future functional validation will be needed to confirm their regulatory interaction with miR-30a and other family members.

Nonetheless, some limitations must be acknowledged. The absence of in vitro and in vivo models prevents the experimental confirmation of mechanistic hypotheses, and the relatively limited sample size, although supported by power calculations, may affect the generalizability of the findings. In particular, subgroup analyses may be underpowered and should be interpreted with caution. Future directions include the functional validation of key targets, the evaluation of non-seed region contributions using anti-miR approaches in OS cell models, and the extension of the analysis to larger, independent cohorts. In addition, expression analysis was conducted on FFPE tissue, where cellular heterogeneity and the lack of spatial resolution may influence results. Serum- or plasma-based validation will also be needed to assess the diagnostic utility of miR-30a and miR-30e as circulating biomarkers.

Despite these limitations, the integrated framework proposed here, combining molecular profiling, regulatory prediction, and histopathological evidence, provides a biologically grounded basis to refine diagnostic and therapeutic strategies in canine OS.

## 5. Conclusions

This study outlines a biologically driven strategy to refine the molecular classification of canine osteogenic tumors. By integrating IHC marker selection with miRNA regulatory profiling, the miR-30 family was identified as a candidate axis with diagnostic utility. Nonetheless, further investigations are warranted to validate these findings in larger cohorts, to explore the functional effects of miR-30a/e via knockdown or overexpression assays, and to assess their diagnostic potential in serum or plasma. Moreover, the potential influence of tissue-specific expression and cellular heterogeneity in FFPE samples should be taken into account in future research. The observed expression patterns and bioinformatic predictions support a coordinated regulatory mechanism involving RUNX2, KPNA2, and SATB2, primarily mediated by miR-30a and miR-30e. Altogether, these findings propose a miRNA-guided approach as a complementary tool to improve diagnostic specificity and support early identification of OS and EOS.

## Figures and Tables

**Table 1:** Case recruitment and histological classification. The table lists the cases selected in the study: EOS (*n* = 19), OS (*n* = 14), and the healthy CTRL (*n* = 10). Age (years), sex, breed, anatomical localization, and histological diagnosis are reported for each subject. Abbreviations: M, male; F, female; NA, data not available.

Breed	Age	Sex	Tumor Localization	Histological Diagnosis
English Cocker Spaniel	14	M	Intestine	EOS-1
Mixed breed	8	F	Mammary gland	EOS-2
Mixed breed	14	F	Mammary gland	EOS-3
Mixed breed	10	F	Mammary gland	EOS-4
NA	9	M	Urinary bladder	EOS-5
Mixed breed	12	F	Spleen	EOS-6
Mixed breed	13	F	Mammary gland	EOS-7
Hunting dog	13	F	Uterus	EOS-8
Mixed breed	8	F	Mammary gland	EOS-9
Mixed breed	12	F	Intestine	EOS-10
Cirneco dell’Etna	15	F	Haired skin (thorax)	EOS-11
English Cocker Spaniel	12	M	Haired skin	EOS-12
Mixed breed	10	F	Mammary gland	EOS-13
Maremma Sheepdog	9	F	Subcutis (shoulder area)	EOS-14
Labrador Retriever	10	M	Thyroid gland	EOS-15
Mixed breed	11	F	Mammary gland	EOS-16
Epagneul Breton	10	F	Subcutis (dorsal area)	EOS-17
Mixed breed	13	F	Mammary gland	EOS-18
English Pointer	11	F	Mammary gland	EOS-19
Rottweiler	11	F	Femur	OS-1
Labrador Retriever	12	F	Humerus	OS-2
Mixed breed	10	F	Radium	OS-3
Mixed breed	7	M	Vertebrae	OS-4
Mixed breed	9	M	Maxilla	OS-5
American Staffordshire Terrier	9	M	Tibia	OS-6
Pinscher	4	F	Femur	OS-7
Pitbull	6	F	Scapula	OS-8
American Stafford	12	F	Front limb	OS-9
Mixed breed	9	M	Carpus	OS-10
Belgian Sheperd	12	M	Mandibula	OS-11
Greyhound	6	F	Humerus	OS-12
German Shepherd	10	F	Forelimb	OS-13
Saint Bernard	9	F	Humerus	OS-14
Mixed breed	9	M	Mandibula	CTRL-1
Rottweiller	11	F	Haired skin	CTRL-2
Briquet Griffon	6	F	Spleen	CTRL-3
Briquet Griffon	6	F	Urinary Bladder	CTRL-4
Mixed breed	1	F	Uterus	CTRL-5
Mixed breed	8	F	Spleen	CTRL-6
Mixed breed	8	F	Humerus	CTRL-7
Mixed breed	15	M	Rib	CTRL-8
Mixed breed	8	F	Subcutis (mammary area)	CTRL-9
Mixed breed	8	F	Intestine	CTRL-10

**Table 2:** MiR-30 family members from canine (cfa), human (hsa), and chicken (gga) species. Columns show, from left to right: the miRNA name with corresponding miRBase v22.1 ID, mature sequence (5′–3′), conserved seed region (nucleotides 2–8), and variable non-seed region (nucleotides 9–22). The seed region is identical among all listed miRNAs, while sequence diversity within the non-seed region may underlie differences in target affinity and regulatory specificity.

miRNA	Mature Sequence (5′–3′)	Seed	Non-Seed (nt 9–22)
		(nt 2–8)	
cfa-miR-30a MIMAT0006604	UGUAAACAUCCUCGACUGGAAG	GUAAACA	AUCCUCGACUGGAAG
hsa-miR-30b-5p MIMAT0000420	UGUAAACAUCCUACACUCAGCU	GUAAACA	AUCCUACACUCAGCU
gga-miR-30c-5p MIMAT0001137	UGUAAACAUCCUACACUCUCAGCU	GUAAACA	AUCCUACACUCUCAGCU
cfa-miR-30d MIMAT0006616	UGUAAACAUCCCCGACUGGAAGCU	GUAAACA	AUCCCCGACUGGAAGCU
hsa-miR-30e-5p MIMAT0000692	UGUAAACAUCCUUGACUGGAAG	GUAAACA	AUCCUUGACUGGAAG

**Table 3:** List of target miRNAs, reference miRNAs, and spike-in controls used in the study. The table includes miRNAs from Canis familiaris (cfa), Homo sapiens (hsa), and Gallus gallus (gga), with their respective miRBase accession numbers and QIAGEN catalog numbers. Primers designed for non-canine species exhibited cross-reactivity with Canis familiaris. Eleven specific primer pairs are included in the study: five target miRNAs (miR-30a, miR-30b, miR-30c, miR-30d, miR-30e), three candidate endogenous miRNA controls (hsa-miR-26a-5p, hsa-miR-103a-3p, hsa-miR-186-5p), and three spike-in controls (UniSp2, UniSp4, and UniSp6). Spike-in control assays are used as exogenous, non-species-specific normalization controls.

MiRNA	Specie Name	miRBase Accession	Qiagen ID
cfa- miR-30a	*Canis familiaris*	MIMAT0006604	YP02101182
hsa- miR-30b-5p	*Homo sapiens*	MIMAT0000420	YP00204765
gga- miR-30c-5p	*Gallus gallus*	MIMAT0001137	YP00205948
cfa- miR-30d	*Canis familiaris*	MIMAT0006616	YP02118689
hsa- miR-30e-3p	*Homo sapiens*	MIMAT0000693	YP00204410
hsa- miR-26a-5p [46]	*Homo sapiens*	MIMAT0000082	YP00206023
hsa- miR-103a-3p [47]	*Homo sapiens*	MIMAT0000101	YP00204063
hsa- miR-186-5p [46]	*Homo sapiens*	MIMAT0000456	YP00206053
UniSp2	All species	None	YP00203950
UniSp4	All species	None	YP00203953
UniSp6	All species	None	YP00203954

**Table 4:** Immunohistochemical evaluation of RUNX2 expression in canine samples. “% Labeled Cells” indicates the proportion of RUNX2-positive nuclei among neoplastic cells. “Score” refers to the semi-quantitative assessment of labeling extent (scale: 0–4), while “Intensity” indicates the nuclear staining strength (scale: 0–3). Superscript letters (a, b) denote statistically defined groups based on Mann–Whitney U tests: values sharing the same letter are not significantly different (*p* > 0.05); values with different letters are significantly different (*p* < 0.05).

Histological Diagnosis	% Labeled Cells	Score	Intensity
EOS-1	30 ^b^	2 ^b^	3 ^b^
EOS-2	70 ^b^	3 ^b^	2 ^b^
EOS-3	80 ^b^	4 ^b^	3 ^b^
EOS-4	60 ^b^	3 ^b^	2 ^b^
EOS-5	90 ^b^	4 ^b^	3 ^b^
EOS-6	40 ^b^	2 ^b^	3 ^b^
EOS-7	40 ^b^	2 ^b^	3 ^b^
EOS-8	80 ^b^	4 ^b^	3 ^b^
EOS-9	50 ^b^	3 ^b^	3 ^b^
EOS-10	75 ^b^	3 ^b^	2 ^b^
EOS-11	90 ^b^	4 ^b^	3 ^b^
EOS-12	30 ^b^	2 ^b^	3 ^b^
EOS-13	70 ^b^	3 ^b^	3 ^b^
EOS-14	25 ^b^	2 ^b^	3 ^b^
EOS-15	50 ^b^	3 ^b^	3 ^b^
EOS-16	35 ^b^	2 ^b^	3 ^b^
EOS-17	40 ^b^	2 ^b^	3 ^b^
EOS-18	15 ^b^	1 ^b^	3 ^b^
EOS-19	50 ^b^	3 ^b^	3 ^b^
OS-1	30 ^b^	4 ^b^	3 ^b^
OS-2	60 ^b^	3 ^b^	2 ^b^
OS-3	60 ^b^	3 ^b^	2 ^b^
OS-4	50 ^b^	3 ^b^	3 ^b^
OS-5	80 ^b^	4 ^b^	3 ^b^
OS-6	30 ^b^	2 ^b^	2 ^b^
OS-7	70 ^b^	3 ^b^	3 ^b^
OS-8	30 ^b^	2 ^b^	2 ^b^
OS-9	40 ^b^	2 ^b^	2 ^b^
OS-10	30 ^b^	3 ^b^	3 ^b^
OS-11	60 ^b^	2 ^b^	2 ^b^
OS-12	70 ^b^	3 ^b^	3 ^b^
OS-13	40 ^b^	4 ^b^	3 ^b^
OS-14	30 ^b^	4 ^b^	3 ^b^
CTRL-1	0 ^a^	0 ^a^	0 ^a^
CTRL-2	0 ^a^	0 ^a^	0 ^a^
CTRL-3	0 ^a^	0 ^a^	0 ^a^
CTRL-4	0 ^a^	0 ^a^	0 ^a^
CTRL-5	0 ^a^	0 ^a^	0 ^a^
CTRL-6	0 ^a^	0 ^a^	0 ^a^
CTRL-7	0 ^a^	0 ^a^	0 ^a^
CTRL-8	0 ^a^	0 ^a^	0 ^a^
CTRL-9	0 ^a^	0 ^a^	0 ^a^
CTRL-10	0 ^a^	0 ^a^	0 ^a^

**Table 5:** RefFinder comprehensive ranking. The table shows the stability value rankings assigned by RefFinder. The RefFinder method provides a comprehensive ranking that categorizes miRNAs as “best,” “good,” or “average” by ordering them from the most stable (miR-103a-3p) to the least stable (miR-186-5p).

Analysis Method			
Delta CT	miR-103a-3p	miR-26a-5p	miR-186-5p
BestKeepeer	miR-103a-3p	miR-26a-5p	miR-186-5p
NormFinder	miR-103a-3p	miR-26a-5p	miR-186-5p
GeNorm	miR-26a-5p/miR-103a-3p	miR-186-5p	
**RefFinder Ranking Order**	**miR-103a-3p**	**miR-26a-5p**	**miR-186-5p**
	Better	Good	Average

**Table 6:** The table reports the AUC with 95% confidence intervals, the optimal cutoff value (identified based on the highest LR^+^), sensitivity, specificity, and LR^+^. The Diagnostic Value column qualitatively classifies the clinical relevance of each LR^+^ as follows: limited or not useful (≤2), acceptable (2–5), good (5–10), or strong (>10) [60]. Comparisons were performed between CTRL and OS or C versus EOS samples (highlighted in grey). Cutoff directionality reflects the expression trend of each miRNA: values below the threshold indicate disease in cases where the cutoff is “<“ (for OS: miR-30a, miR-30b, miR-30c, miR-30d, and miR-30e; for EOS: miR-30a, miR-30b, and miR-30e), whereas values above the threshold indicate disease when the cutoff is expressed as “>“ (for EOS: miR-30c).

	miRNA	AUC (IC 95%)	Cutoff	Sensitivity (%)	Specificity (%)	LR^+^	Diagnostic Value
CTRL vs. OS	miR-30a	0.714	<0.280	71.43	85.71	5	Good
	miR-30b	0.780	<1.184	60.00	80.00	3	Acceptable
	miR-30c	0.614	<0.587	50.00	85.71	3.5	Acceptable
	miR-30d	0.518	<0.419	40.00	83.33	2.4	Acceptable
	miR-30e	0.900	<0.094	70.00	90.91	7.7	Good
CTRL vs. EOS	miR-30a	0.691	<0.280	66.67	85.71	4.7	Acceptable
	miR-30b	0.631	<1.184	46.15	80.00	2.3	Acceptable
	miR-30c	0.774	>1.225	50.00	85.71	3.5	Acceptable
	miR-30d	0.667	>0.614	58.33	83.33	3.5	Acceptable
	miR-30e	0.762	<0.093	61.54	90.91	6.8	Good

## Data Availability

The data supporting the findings of this study are available in the Supplementary Material.

## Supplementary Materials

The following supporting information can be downloaded at: https://www.mdpi.com/article/10.3390/cells14161279/s1: Table S1: Computational prediction of interactions between miRNAs belonging to the miR-30 family (miR-30a, miR-30b, miR-30c, miR-30d, miR-30e) and selected target genes (RUNX2, SATB2, and KPNA2) based on Target Scan (Context++ Score/PCT), miRDB (Target Score), RNA22 (Sites/Energy), and RNA hybrid (Min Free Energy). Table S2: Quantification of miR-30 family members in OS, EOS and CTRL samples.
