# Supplementary material for: A miR-30 Guided Molecular Profiling of Canine Osteosarcoma and Extraskeletal Osteosarcoma Reveals Non-Seed Regulatory Divergence
Source: Cells. 2025 Aug 18;14(16):1279. doi: 10.3390/cells14161279 (PMC12384767; doi:10.3390/cells14161279)
Supplement: Supplementary file 1 [file cells-14-01279-s001.zip › cells-3749531-supplementary.pdf]

Table S1

Computational prediction of interactions between miRNAs belonging to the miR-30 family (miR-30a, miR-30b, miR-30c, miR-30d, miR-30e) and selected target genes (RUNX2, SATB2, and KPNA2) based on TargetScan (Context++ Score / PCT), miRDB (Target Score), RNA22 (Sites / Energy), and RNAhybrid (Min Free Energy) . TargetScan results are presented as Context++ scores combined with the probability of conserved targeting (PCT). Lower Context++ scores (more negative) suggest stronger predicted repression, while higher PCT values indicate greater evolutionary conservation of the binding site. miRDB scores range from 50 to 100, with higher scores reflecting greater confidence in the predicted interaction. RNA22 predicts the number of binding sites and their combined binding energy (in kcal/mol), with more sites and lower energies suggesting stronger binding. RNAhybrid reports the minimum free energy of the miRNA-mRNA duplex (in kcal/mol), with lower values indicating more stable binding.

miR-30 Family	Target Gene	TargetScan	miRDB	RNA22	RNAhybrid
miR-30a	RUNX2	–0.22 / 0.80	96	2 sites / –23.0 kcal/mol	–26.0 kcal/mol
miR-30a	SATB2	–0.18 / 0.66	65	1 site / –20.1 kcal/mol	–23.2 kcal/mol
miR-30a	KPNA2	–0.18 / 0.69	75	2 sites / –21.3 kcal/mol	–24.5 kcal/mol
miR-30b	RUNX2	–0.22 / 0.80	96	2 sites / –21.7 kcal/mol	–24.0 kcal/mol
miR-30b	SATB2	–0.18 / 0.66	65	1 site / –19.4 kcal/mol	–22.9 kcal/mol
miR-30b	KPNA2	–0.19 / 0.69	75	2 sites / –20.8 kcal/mol	–23.8 kcal/mol
miR-30c	RUNX2	–0.22 / 0.80	96	2 sites / –21.0 kcal/mol	–24.1 kcal/mol
miR-30c	SATB2	–0.18 / 0.66	65	1 site / –18.7 kcal/mol	–22.3 kcal/mol
miR-30c	KPNA2	–0.19 / 0.69	75	2 sites / –20.3 kcal/mol	–23.6 kcal/mol
miR-30d	RUNX2	–0.22 / 0.80	96	2 sites / –22.1 kcal/mol	–25.0 kcal/mol
miR-30d	SATB2	–0.18 / 0.66	65	1 site / –19.0 kcal/mol	–22.7 kcal/mol
miR-30d	KPNA2	–0.18 / 0.69	75	2 sites / –20.6 kcal/mol	–24.3 kcal/mol
miR-30e	RUNX2	–0.20 / 0.80	96	2 sites / –22.5 kcal/mol	–25.6 kcal/mol
miR-30e	SATB2	–0.18 / 0.66	65	1 site / –20.0 kcal/mol	–23.4 kcal/mol
miR-30e	KPNA2	–0.19 / 0.69	75	2 sites / –21.7 kcal/mol	–25.1 kcal/mol

Table S2

Quantification of miR-30 family members in OS, EOS and CTRL samples. Fold changes were calculated using the 2^–ΔΔCq method, which involves: (1) Calculation of ΔCq values by subtracting the reference Cq (miR-103a-3p) from the target Cq for each individual sample; (2) calculation of ΔΔCq relative to the calibrator group (CTRL, n = 10), where ΔΔCq = 0 corresponds to $2^0 = 1$; and (3) transformation to relative expression as 2^–ΔΔCq. The table reports, for each miRNA: the mean Cq ± SEM for both target and reference; ΔCq values normalized to the reference gene; 2^–ΔCq as a measure of normalized expression; ΔΔCq calculated using the control group as calibrator; and the resulting fold change (2^–ΔΔCq) representing relative expression in the OS and EOS groups. Samples belonging to the same experimental group are averaged by the StepOne™ Software v2.3 (Applied Biosystems), according to the plate layout set on the StepOnePlus™ Real-Time PCR System. Data are exported as mean ± SEM. If the software or the operator detects outliers or unusually high SEM values, individual sample replicates are reviewed and analyzed separately.

	OS (mean ± SEM; n = 14)					
miRNA	Cq Target	Cq reference	ΔCq	2^−ΔCq	ΔΔCq	Fold changes
miR-30a	28.400 ± 1.144	26.590 ± 1.316	1.810 ± 0.650	0.285 ± 0.093	0.336 ± 0.770	0.821 ± 0.514
miR-30b	27.341 ± 0.697	27.256 ± 0.720	0.085 ± 0.180	0.943± 0.116	0.722 ± 0.182	0.606 ± 0.075
miR-30c	27.890 ± 0,652	27.518 ± 0.740	0.372 ± 0.350	0.773 ± 0.184	0.060 ± 0.344	0.959 ± 0.228
miR-30d	28.425 ±0.338	27.310 ± 0.479	1.115 ± 0.340	0.462 ± 0.11	0.129 ± 0.348	0.914 ± 0.219
miR-30e	31.825 ± 0.565	28.065 ± 0.644	3.760 ± 0.310	0.074 ± 0.076	1.786 ± 0.321	0.290 ± 0.064

	EOS (mean ± SEM; n = 19)					
miRNA	Cq Target	Cq reference	ΔCq	2^−ΔCq	ΔΔCq	Fold changes
miR-30a	30.487 ± 0.984	30.007 ± 1.152	0.480 ± 0.600	0.717 ± 0.298	−0.459 ± 0.599	1.375 ± 0.572
miR-30b	28.579 ± 0.677	29.287 ± 0.729	−0.708 ± 0.270	1.632 ± 0.305	−0.070 ± 0.271	1.050 ± 0.166
miR-30c	28.097 ± 0.437	29.009 ± 0.527	−0.912 ± 0.295	1.879 ± 0.284	−1.222 ± 0.293	2.330 ± 0.477
miR-30d	29.356 ± 0.355	29.315 ± 0.471	0.041 ± 0.310	0.972 ± 0.209	−0.942 ± 0.310	1.921 ± 0.412
miR-30e	32.465 ± 0.451	30.489 ± 0.808	1.976 ± 0.670	0.254 ± 0.020	0.020 ± 0.672	0.986 ± 0.456

	Control (mean ± SEM; n = 10)			
miRNA	Cq Target	Cq reference	ΔCq	2^−ΔCq
miR-30a	25.975 ± 2.740	25.155 ± 2.749	0.820 ± 0.222	0.566 ± 0.087
miR-30b	25.186 ± 2.653	25.857 ± 2.657	−0.671 ± 0.153	1.592 ± 0.169
miR-30c	25.957 ± 2.714	25.779 ± 2.724	0.178 ± 0.235	0.886 ± 0.144
miR-30d	26.947 ± 2.833	25.986 ± 2.835	0.961 ± 0.110	0.513 ± 0.039
miR-30e	28.198 ± 3.211	26.666 ± 3.226	1.532 ± 0.313	0.346 ± 0.035
